# Strategies to Enhance Drug Permeability across Biological Barriers—A Summary of This Important Special Issue

**DOI:** 10.3390/pharmaceutics15041189

**Published:** 2023-04-08

**Authors:** Jingyuan Wen, Yuan Huang

**Affiliations:** 1 The School of Pharmacy, Faculty of Medical Health Science, University of Auckland, Auckland 1023, New Zealand; 2 West China School of Pharmacy, Sichuan University, Chengdu 610093, China

This Special Issue, “Strategies to Enhance Drug Permeability across Biological Barriers”, is hosted by Pharmaceutics and highlights the recent technological advancements for overcoming biological barriers and improving drug permeability and absorption.

The delivery of therapeutic drugs to desired sites at required rates/extents is limited by the numerous biological barriers in the body, including the intestinal epithelium membrane, blood–brain barrier (BBB), and skin barrier. The drug permeability of almost all macromolecular drugs and most small lipophilic drugs, across the intestinal membrane, for instance, will be severely limited by the physical barriers that are presented by the mucous layer, the epithelial membrane, and the tight junctions, in addition to the enzymatic barrier for unstable compounds. Experts in the drug delivery field need to find advanced, non-invasive methods to persistently overcome these biological barriers. This Special Issue highlights the recent advancements in overcoming these barriers and improving drug permeability. The 11 papers in this Special Issue, contributed by global experts to explore various advanced strategies, provide new insights on all aspects of the enhancement of drug penetration across biological barriers.

The first article of this Special Issue looks at the recent advances in cancer-targeting drug research, which have been made by overcoming the multiple biological barriers that are associated with mitochondrial targeting. Yi et al. established a 2-(dimethylamino) ethyl methacrylate (DEA)-modified N-(2-hydroxypropyl) methacrylamide (HPMA) copolymer–CPT conjugate (P-DEA-CPT) to mediate the mitochondrial accumulation of CPT [1]. This mitochondria-targeting P-DEA-CPT can quickly internalize into 4T1 cells, escaping from lysosomes and accumulating inside mitochondria. P-DEA-CPT has shown the successful in vivo inhibition of metastasis with decreased side effects, suggesting mitochondrial targeting as a promising treatment of metastatic tumors.

Similarly, Zhang et al. managed to overcome the barriers of cancer cells by using cholera toxic subunit b (CTB) [2]. A more significant in vivo targeting of lung metastasis, via a chemical conjugation of a CTB protein onto the surface of PEGylated liposomes (CTB-sLip), was observed when compared to unmodified PEGylated liposomes (sLip).

Li et al. tackled the skin barrier by developing a niosomal nano carrier system to deliver green tea catechins and their analogue, epigallocatechin gallate (EGCG), to the dermis layer, in order to achieve a greater antioxidant effect [3]. In this study, a drug-loaded niosomal delivery system delivered EGCG to the dermal layer and effectively prolonged its release, demonstrating a much deeper skin penetration and deposition than free EGCG. The resulting antioxidant effects, in comparison to those of free EGCG, were seen through an increased cell survival after UVA irradiation, a reduced lipid peroxidation, and increased antioxidant enzyme activities in the human dermal fibroblasts (Fbs).

Eedara et al. reviewed the application of dry powder inhalation to treat lung diseases such as asthma, cystic fibrosis, and lung infections, by crossing the lung barrier [4]. Due to a lack of a standard dissolution methods and absorption models, this review looked into various dissolution systems and evaluated their performances in crossing the lung barrier.

Ryall et al. reviewed the approaches and techniques from the last two decades for the topical delivery of wound-healing bioactives [5]. Many natural products have desirable biological properties that are applicable to wound healing, but are limited by their inability to cross the stratum corneum to access the wound. Such natural products and their reapplication, through the use of modern delivery methods such as niosomes and microneedles, were summarized in this review. The molecular mechanism of wound healing was also examined in detail.

He et al. reviewed our understanding of membrane proteins and drug permeability by editing the expression of these membrane proteins using the CRISPR-Cas9 system, which is the most developed and used CRISPR-associated Cas system [6]. Both methods of genome editing that use CRISPR-Cas9 and their applications for improving our understanding of biological barriers were compiled in this paper.

There are many ways to overcome biological barriers and deliver a drug to its intended location. Crowe and Hsu compiled the recent research on completely bypassing the BBB through the intranasal route, which will be an essential step towards the treatment of patients with neurological diseases [7]. Methods for improving the safety and efficacy of intranasal formulations were reviewed in this paper, including the use of nasal permeability enhancers, gelling agents, and nanocarrier formulations.

Alternatively, another way to overcome biological barriers is to increase their permeability through a conjugation with lipid moieties. Tran et al. summarized the cellular internalization effects of hydrophobic moieties that were bound to oligonucleotides [8]. The common hydrophobic moieties in this paper included fatty acids, cholesterol, tocopherol, and squalene. Tran et al. also looked into the clinically successful oligonucleotide conjugates that are currently in use.

The final three reviews looked into the advances in conquering the BBB. Han reviewed the multitude of methods that are currently used to modulate the BBB’s permeability, such as opening tight junctions, inhibiting active efflux, and/or enhancing transcytosis [9]. This review outlined the strategies, mechanisms, and safety of such BBB permeability modulators for a better pre-clinical and clinical study design.

Rechberger et al. bibliometrically summarized the efforts and trends of intra-arterial therapy for brain tumors that have emerged over the past several decades [10]. This bibliographical review of the recent clinical trials and publications in this field of research aimed to provide an idea of the future trends in this field.

Finally, Gandhi et al. took an in-depth dive into the modulation of the BBB’s permeability by using an ultrasound to deliver normally impenetrable drugs into the brain [11]. This review featured information from diverse ultrasound parameters that had already used to achieve an increase in the BBB’s permeability. Microbubbles, transducer frequency, peak-negative pressure, pulse characteristics, and the dosing of ultrasound applications were also included in this review. In total, 107 articles and their protocols, parameters, safety, and efficacy were identified and summarized to help in achieving the standardization of these protocols and parameters in future preclinical and clinical studies.

This special issue features a comprehensive array of approaches and technologies developed to deliver drugs through a variety of biological barriers. These articles showcased in this issue illuminate the challenges posed by these barriers and provide insight into the latest advanced technologies used to overcome them. We are confident that the content of this issue will prove informative and engaging to readers, and we extend our sincere appreciation to all contributors who have made this special issue possible. Thank you for your interest in Pharmaceutics, and we hope you find this special issue enlightening.
